# Intergenerational Factors Influencing Household Cohabitation in Urban China: Chengdu

**DOI:** 10.3390/ijerph18084289

**Published:** 2021-04-18

**Authors:** Meimei Wang, Yongchun Yang, Mengqin Liu, Huailiang Yu

**Affiliations:** 1 School of Resource and Environmental Science, Lanzhou University, Lanzhou 730000, China; wangmm@lzu.edu.cn; 2 College of Environment and Resource, Southwest University of Science and Technology, Mianyang 621010, China; lmq4336@163.com; 3 College of Water Conservancy and Architecture Engineering, Tarim University, Xinjiang 843300, China; yuhuailiang11@foxmail.com

**Keywords:** household habitation, elder, intergenerational factors, relations, Chengdu

## Abstract

Family composition impacts individual consumption habits, which may potentially transform urban integral space structure. Due to the reform in the housing system at the end of the 1990s and increases in residents’ income, houses became more affordable, and intergenerational household cohabitation is no longer the primary pattern. Nonetheless, as families change, it still remains an important form of family composition. Intergenerational support is important in household habitation. This study examines the temporal changes and the structure of intergenerational household cohabitation. Moreover, intergenerational factors in groups of all genders and ages are analyzed. We found that intergenerational household cohabitation in Chengdu comprises three structures: elders living with married children, elders living with unmarried children, and elders living with grandchildren. According to multiple logistic regression, we can see that inadequate housing, economy of costs, cases of emergency, fear of loneliness, care of grandchildren, and poor health have marked effects on household cohabitation, and the positive or negative effects are distinct regarding different structures. To be more specific, the significance of financial support in family composition decreases, and that of support in daily care increases with age. The influence of financial support, daily care support, and emotional support peaks among those aged between 35–60, followed by individuals under 35, and those aged over 60. Financial support is comparatively important for individuals under 35, and females attach more importance to emotional support in intergenerational household cohabitation. The findings provide a basis for subsequent studies of family composition.

## 1. Introduction

Household cohabitation is defined as a residential behavior pattern and if two generations with blood ties live and reside together under the same roof, this is a continuation of Chinese traditional family values, signifying filial piety. In this economic and cultural transition period, Chinese families are undergoing similar changes as Western families are. Nonetheless, since eastern countries are different from western countries in terms of economy, technology, policy, law and socio-cultural background, they have their peculiar features regarding family composition. Before the founding of New China in 1949, extended families with several generations living together typically dominated Chinese society, in which case parents raised their children, arranged their children’s’ marriage, acted as babysitters, and were supported by young couples [1]. Thus, Chinese traditional families usually involved several generations in one household. Housing resources were deficient in the planned economy in the 1950s, and urban residents were paid extremely low wages in that period [2].Residents had no opportunity to choose a house due to the housing system, i.e., the working unit system [3]. Deng’s reforms and opening-up policy in 1978 promoted western culture in mainland China which brought changes to Chinese traditional cultural values, which were refactored [4]. With improvements in the household economy, small families predominated in family composition. Nonetheless, as families changed, household cohabitation became a specific form of family pattern in particular periods in urban China.

**Figure 1:**
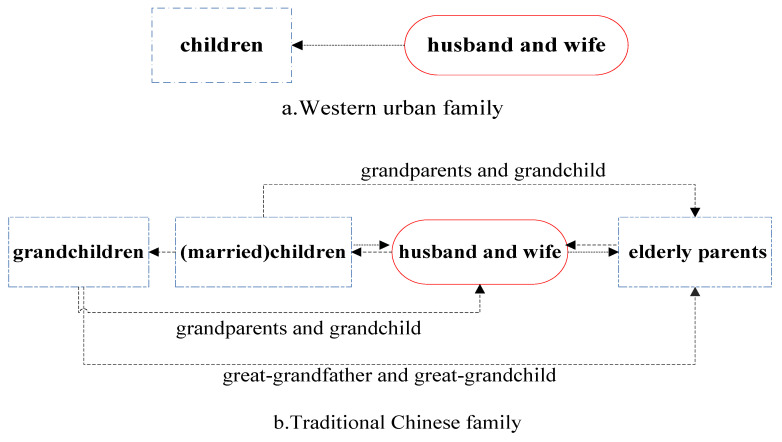
Intergenerational relationships in Chinese and Western families.

The family is the basic unit of social life involving blood and marital relationships [5], and its composition demonstrates different patterns. In China, elders traditionally live together with their children, thus extended families occur where lineal relatives by blood live together. Often with four generations under one roof, which is typical of Chinese traditional cohabitational culture. The head of household (usually parents or grandparents) chose a house located close to schools. However, young people usually chose to move out and select an apartment near their workplace once they were financially capable of doing so. When their parents get older and retire, these offspring choose to live near their parents’ home or live with their parents again [6]. Thus, family composition changes in synchrony with the family life cycle and individuals’ life stages. This means that research into family composition based on intergenerational support is of great importance. Intergenerational cohabitation is the embodiment of traditional Chinese culture, and also reflects a different intergenerational familial relationship compared to that in the West [7]. Western households are generally composed of parents and children; however, the traditional Chinese family is composed of parents and children and grandchildren or grandparents [8], as shown in Figure 1.

**Figure 2:**
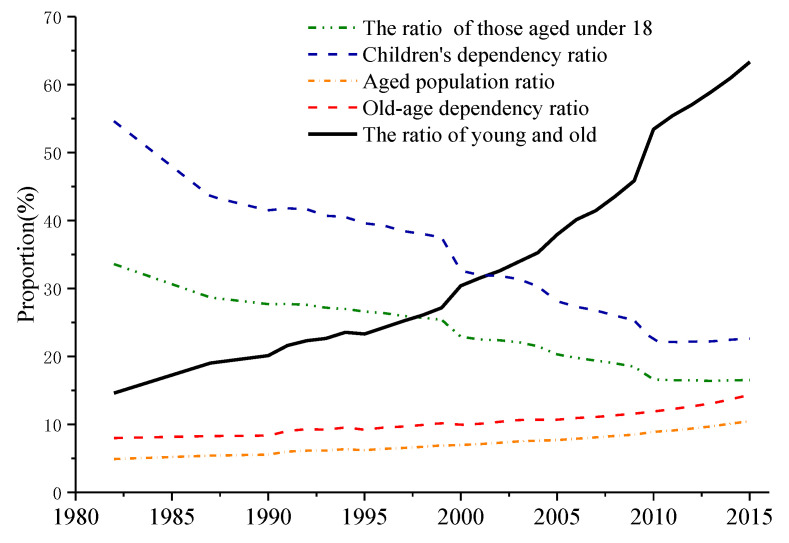
Aging population in China.

In 1982, the International Conference on Aging in Vienna published the Vienna Declaration and Programme of Action, in which problems of aging were bifurcated into humanitarian issues [9]. The family structure an important aspect of society [10]. Family composition impacts individual consumption habits, which may potentially transform urban integral space structure. To date, research into intergenerational support [10,11] based on family ties has mainly focused on elder care [12], concerning their well-being [13], living arrangements [14], traditional values [15], and functional support [16,17,18]. Family-support and migration in urban areas depends on family ties [19]. Demographic transition is a gradual process, taking about 150 years in developed countries [20]. However, demographic transition in China was completed within a short period of time [1] influenced by the underdeveloped economy and the birth-control policy [21]. By the third population census in 1982, the ratio of those aged under 18 (i.e., ratio of the population under 18 in the total population) amounted to 33.59%, the children’s dependency ratio (which compares the number of children with those aged 15–64) was 54.61%, the aged proportion ratio (i.e., the mean ratio of the proportion of the aging population in the total population) was 4.91%, the old-age dependency ratio (which compares the number of people over 65 with those aged under 18) was 7.98%, and the ratio of young and old (which compares the number of people over 65 with those aged 15 to 64) was 14.62%. In 2015, these figures changed to 16.52%, 22.63%, 10.47%, 14.33%, and 63.33%, respectively. Thus, it can be seen that the proportion of those aged under 18 and the child dependency ratio experienced a sharp decline over 33 years, while the proportion of elders, the old-age dependency ratio, and the ratio of young to old increased rapidly (Figure 2). In other words, China’s aging population has become more serious. In the face of the trend towards an aging population, these topics deserve attention in further research.

The intergenerational relationship in urban families in China is akin to the traditional Chinese family, but it is not consistent during the transformation period. From a macroscopic view, intergenerational cohabitation determines the location of residency, social humanistic environment, and infrastructure. From a microcosmic view, it also influences the choice of family composition. The intergenerational residential mode and housing choices based on the family life cycle are closely related to the trend of family miniaturization, as occurs in modern Western society and traditional cohabitation culture. Starting with support between generations, this article analyzes Chinese urban intergenerational habitation based on traditional concepts. Data were collected by means of extensive face-to-face interviews in Chengdu city from 2016 to 2018. In the process of modernization in mainland China, household cohabitation is of profound social and cultural significance. First, it integrates upbringing and support, which helps alleviate pressure on young couples. Second, it is a suitable example of the situation in which China is getting old before it has got rich. In brief, household cohabitation effectively maintains social stability in China by relieving pressure in house-purchasing, child-rearing, and supporting the elderly within a family. Hence, it is necessary to explore family composition for urban citizens in the mid-western cities of China from the viewpoint of traditional Chinese customs and culture. We seek to answer the following questions. First, does household cohabitation still exist during the transition period? Second, in what form do elders in cities cohabit with their descendants? Third, which intergenerational factors have influence on different forms of household cohabitation?

## 2. Literature Review

Family life cycle was first proposed by Rowntree [22], defined by the relations between income and demand. This offered a paradigm from which we can study household consumption. From studies of family composition associated with Rossi’s seminal study [23], research on family composition in Western contexts has been enriched, and the theoretical model has been deepened and perfected. Moreover, the methodology used in studying the family life cycle became the core methodology for research on family composition. The life cycle consumption model and the life cycle savings hypothesis were presented and the general equilibrium model of family life cycle consumption was presented. The optimal choice of structure and mode of reality for labor income and family life cycle prices was also provided. In addition, it has been shown that trends in support for elders varies between generations [24]. Furthermore, factors impacting intergenerational support have also been studied for specific groups [25], such as elder couples without children, and migrants.

In 1865, France became the world’s first aging country, followed by other European countries such as Sweden. Subsequently, these countries have paid great attention to family composition and its influencing factors. From the late 18th century, the patriarchal colonial family gradually began to shift toward the more self-reliant, democratic, and affectionate family pattern some historians characterize as the “modern family” [26]. The major shift in the family structure of the older American population occurred during the 20th century, especially in the period since World War II. This transition to living alone came long after discontinuities had developed in other major indicators of economic, demographic, familial and attitudinal modernization [27]. Industrialization was an important cause of change, as it brought about the detachment of productive work from the home [28].

Western and Eastern scholars have conducted extensive research pertaining to families. Western scholars have paid more attention to the microscopic study of family care, organizational support, and elders’ health, etc. [29]. Moreover, deeper explorations have been conducted from the perspectives of housing patterns, residential mobility [30] and so on. Diversified family modes have spread throughout the late 20th century [31], and thereby influenced residents’ decisions regarding caring for the elderly, which has predominated in research contexts [32]. In addition, research has covered aspects such as the diversification of elders’ support systems [33], senior care [34], and systems for elderly care services [35,36]. Of course, the marital status of children affects the intergenerational connection [37]. In brief, generational studies rely on analyses of stylized versions of family systems, namely the conjugal, stem and joint systems [38]. Family composition for the elderly shows marital and regional differences [39].

Guangdan (1936) carried out a social investigation into the family system, finding that family ties maintain family endowment [10]. Comprehensive research into intergenerational support in China started in the early 1980s, and existing studies on the care of urban elder residents in China have mainly concentrated on the definition and classification of old-age support patterns [40,41], the wish to choose a particular kind of support model [42], and factors involved in choosing particular models [43,44]. Family composition shows a series of attributes [45]. It is unclear how urban families in China determine their household composition in this economic and cultural transition period [46]. Age, gender, marriage status, education [47], and income all affect choices on family composition [48,49]. In addition, Jiang indicated that earning sources and number of children play a part in family composition [50]. In fact, the situation is similar across Asia. The number of children and marriage status are influential factors determining independent living arrangements for the elderly [51,52]. Meanwhile, there is a great discrepancy between the urban elderly and rural elderly [53]. Income only is significant for the urban elderly, but not for the rural elderly. Factors such as education, age, and number of children are significantly related to living arrangements for the rural elderly. For the rural aged, regardless of their marital status, age and number of children are related to separate living arrangements, but income is significant for the aged living in urban areas [54].

Certainly, the Chinese economy has developed at an unprecedented rate in the transitional period. Correspondingly, real estate has prospered. Hence, urban families in China now have more options regarding family composition. Thus, factors that influence family composition in Chinese urban areas are leading to immense diversification. Family composition in China is influenced by the unique Chinese family life cycle, the market economy, and the traditional redistribution system, which demonstrate highly complex traits for research purposes. Housing marketization in China started later than that in developed countries. Thus, the government lacks data from longitudinal surveys on individuals and families, which leads to insufficient studies on Chinese family composition. Some scholars from Hong Kong and Western countries did research on family composition in China, and found that Chinese family composition is impacted not only by the different characteristics of individuals and families, but also by their work unit. Huang proposed that social contact between family members, as well as government regulations and the properties of the work unit, significantly impact family composition [55]. Li found that employment-related variables, such as householders’ positions and their workplaces, significantly affect their purchase of a house as a commodity [56] (the commodity house or commercial housing emerged in China in the 1980s, and refers to the market economy; it refers specifically to houses approved by the relevant government departments and developed by real estate and management companies, including residential houses, commercial houses and other buildings, which are sold and rented on the market after completion). In the transformation period, Western cultural values spread and gained recognition among urban senior citizens in China, while the impact of China’s traditional cultural values remains deep-rooted, and the choice of family composition in urban China is complex. First, existing studies on factors related to family composition are mainly empirical, which entail modeling by collecting data and analyzing the influencing factors on the basis of the results of the model. Thus, most are simple statistical descriptions, rather than in-depth analyses. Second, targeted research has been conducted on the impact of personal attributes, such as age or gender. However, systematic summary studies of intergenerational support and family composition are insufficient, and the relative effects of these factors have not yet been compared. Furthermore, research into family composition and its factors has centered on the eastern provinces of China.

## 3. Research Design

### 3.1. Sample and Study Setting

The main metropolitan areas of Chengdu City—Qingyang District, Jinjiang District, Jinniu District, Chenghua District, and Wuhou District—were selected for this study for three reasons. First, there has been rapid development in elderly care due to the significantly aging population [20]. Second, these areas have been subject to a high degree of globalization; in terms of economic aggregate, Chengdu is third among Chinese cities following Guangzhou and Shenzhen. Third, Chengdu has a far-reaching traditional culture, along with a relatively long period of economic and cultural prosperity. For this study, all the interviewees were residents in Chengdu, aged 18–90, and live with at least one adjacent generation in the same accommodation. It is important to note that our sample crosses all age groups, not just older people.

### 3.2. Data Collection

Data were collected via four extensive, in-depth interviews and questionnaire surveys in Chengdu for the periods from September to November 2016, and from June to October in 2018. A total of 4000 copies of the questionnaire were disseminated. Unqualified examples (1535) were removed, including incomplete answers, incomplete personal information, and contradictory answers. Finally, 2465 questionnaires qualified for study (Table 1).

On the basis of the existing literature, we designed the prototype of the questionnaire, and made a small number of in-depth interviews in Chenghua District. Thus, we obtained information on the possible intergenerational factors influencing household cohabitation in Chengdu City. According to the results, the questionnaire survey and questions for in-depth interviews were modified. Thereupon, a small range of tests and in-depth interviews was again conducted in Chenghua District. Having combined the information obtained up to this point, the questionnaire was revised again. Finally, the established questionnaire was disseminated across the five districts of Chengdu City.

According to the sixth census data for 2010, elders (60+) account for 12.91% (856,000) of the total population of Chengdu City. While conducting the large-scale questionnaire survey, different groups of older people in the five districts of Chengdu City were investigated, according to the population ratio. Meanwhile, our research team randomly conducted in-depth interviews in the city’s five districts.

## 4. Results

### 4.1. Statistical Analysis

By the year 2000, the total number of homes in Chengdu was 3,317,526, among which homes incorporating senior citizens accounted for 30.47% (1,010,944), families incorporating one elder 20.92% (694,113), families incorporating two elders 9.42% (312,520), and families incorporating more than two elders 0.13% (4311). In 2010, the total number of homes was 4,547,109 in Chengdu, among which those incorporating senior citizens accounted for 15.69% (713,581), families incorporating one elder 11.93% (542,587), families incorporating two elders 3.71% (168,736), and families incorporating more than two elders 0.05% (2258). The ratio of each mode of intergenerational household cohabitation in the five districts changed (Table 2).

### 4.2. Spatial and Temporal Changes in Intergenerational Household Cohabitation from 2000 to 2010

The number of people and homes had increased markedly in 2010 compared with 2000, nonetheless, families living with senior citizens had decreased drastically. The population increased significantly in Wuhou district (to 552,840 individuals, 226,608 homes), followed by Jinniu district (277,972 individuals, 125,662 homes). The district with the least change was Chenghua district (205,511 individuals, 100,245 homes).

From 2000 to 2010, the decrease in families with an elder was most significant in Wuhou district (decrease of 15,765 homes), followed by Chenghua district, (12,146 homes), and Jinjiang district (7409 homes). The decrease in families with two elders was most obvious in Wuhou district (decrease of 16,795 homes), followed by Chenghua district (12,637 homes). The decrease of families with more than two elders was most prominent in Jinjiang district, where only 205 families had more than two elders, representing a decline over the 10 years.

### 4.3. Distribution of Intergenerational Household Cohabitation According to Age

Intergenerational household cohabitation in Chengdu comprises three structures: (1) elder lives with married children, (2) elder lives with unmarried children, and (3) elder lives with grandchildren. The survey shows that the group of married children living with an elder mainly comprises those aging 25–34, accounting for 14.36% (354), followed by those aged 35–44 at 12.29% (303). With regard to individuals aged under 25 or older than 85, almost none fall into the group of married children living with an elder. Regarding elders with unmarried children, those aged 35–44 account for 7.79% of these children (192), followed by those under 25 accounting for 3.73% (92). Those over 85 rarely fall into the group of unmarried children living with an elder. With respect to the group of elders living with grandchildren, those aged 65–74 accounted for 7.34% (181), followed by those aged 55–64 at 4.38% (108). Again, those older than 85 rarely fall into the group of grandchildren living with an elder.

### 4.4. Intergenerational Factors in Household Cohabitation

#### 4.4.1. Intergenerational Factors in Household Cohabitation

Intergenerational support is the help provided by other members of the family, which may include financial support, daily care, or emotional support. Intergenerational support is one of the informal resources needed to meet the basic requirements of the elderly (Table 3).

#### 4.4.2. Descriptive Statistics

We use an α benchmark of 0.65 for the statistical tests. Table 4 presents the descriptive statistics of the samples. Saving costs, fear of loneliness, and care of grandchildren have a mean of 2.5+, and doing housework has a mean of 1.845. The standard deviation of each factor is between 1.062 and 1.604, showing that the sample is adequate (Table 4).

#### 4.4.3. Multiple Logistic Regression Results

The group of elder and married children acts as a reference group, and the relationship between intergenerational support is then shown for the elderly living with unmarried children, and the elderly living with grandchildren, respectively. Table 5 lists multiple logistic regression results of intergenerational support in household cohabitation. Inadequate housing, saving costs, in case of emergency, fear of loneliness, care of grandchildren, and poor health have marked effects on household cohabitation. It is apparent that:

(1) Inadequate housing has a significant impact on the group of elderly living with unmarried children, and elderly living with grandchildren, as expected, with a positive effect on the former (B = 0.612) and a negative effect on the latter (B = −0.861). Thus, in the face of inadequate housing, the elderly are inclined to live with unmarried children or married children.
(2) Saving costs has a positive effect on the group of elderly living with grandchildren (B = 0.346), and the group of elderly living with unmarried children (B = 0.717); i.e., in order to save costs, the elderly will choose to live with unmarried children or grandchildren.
(3) “In case of emergency” has a negative effect on the group of elderly living with grandchildren (B = −0.518), and a positive effect on the group of elder living with unmarried children (B = 0.221); i.e., people want to have someone to take care of them in case of emergency, so the elderly prefer to live with their unmarried or married children.
(4) Enjoyment of living with a big family does not have a significant impact on the group of elderly living with grandchildren, but has a negative effect on the group of elderly living with unmarried children (B = −0.376). Influenced by the “four generations under one roof” typical of Chinese traditional culture, families enjoy having a large family, so the elderly like to live with their married children.
(5) Doing the housework does not have a significant impact on the group of elderly living with grandchildren, but has a positive effect on the group of elderly living with unmarried children (B = 1.087). That is, in order to get help with doing the housework, the elderly like to live with their unmarried children.
(6) Fear of loneliness has a positive effect on the group of elderly living with grandchildren (B = 0.600), and a negative effect on the group of elderly living with unmarried children (B = −0.300). Thus, in order to avoid loneliness, the elderly like to live with their unmarried or married children.
(7) Care for grandchildren has a positive effect on the group of elderly living with grandchildren (B = 0.471), and has no significant impact on the group of elderly living with unmarried children. Thus, when it comes to taking care of grandchildren, elders elect to live with their grandchildren.
(8) Poor health has a positive effect on the group of elderly living with grandchildren (B = 0.418), and the elderly living with unmarried children (B = 0.239). That is, in order to look after elder relatives who are in poor health, the elderly like to live with grandchildren, or unmarried children.

Hence, inadequate housing, saving costs, in case of emergency, fear of loneliness and poor health are common elements of family inter-generational living. According to factor classification in Table 3, we can say that financial support and daily care support have a greater influence than emotional support on household cohabitation in Chengdu.

#### 4.4.4. Variation in Intergenerational Factors for Different Groups

(1) Variation between different age groups

**Figure 3:**
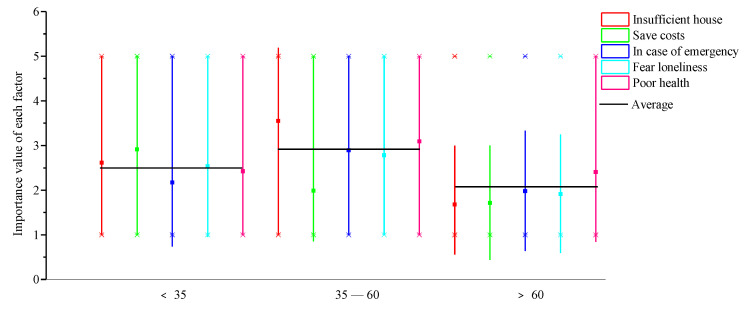
Intergenerational factors in different age groups.

As far as family composition is concerned, intergenerational factors show homogeneity in people of a similar age, while difference is distinct in different age groups. Intergenerational factors of household cohabitation in different age groups are presented in Figure 3. There is considerable difference in intergenerational factors between individuals of different age on the basis of the minimum (Min), the median (Median), the maximum (Max) and the average of the following figures:

First, individuals under 35 seem to worry more about inadequate housing and saving costs, compared to the other age groups. This indicates that financial support is more important when it comes to family composition for individuals under 35.

Second, for those aged between 35–60, inadequate housing and poor health are important considerations in living arrangements. Thus, financial support and daily care are relatively more important than emotional support.

Third, poor health is more critical for individuals aged over 60 years, indicating that daily care counts most for this age group.

The significance of financial support in family composition decreases, and that of support in daily care increases with age, while emotional support is relatively unimportant. Furthermore, the influence of financial support, daily care support, and emotional support peaks among those aged between 35–60, followed by individuals under 35, and those aged over 60.

(2) Variation in gender

**Figure 4:**
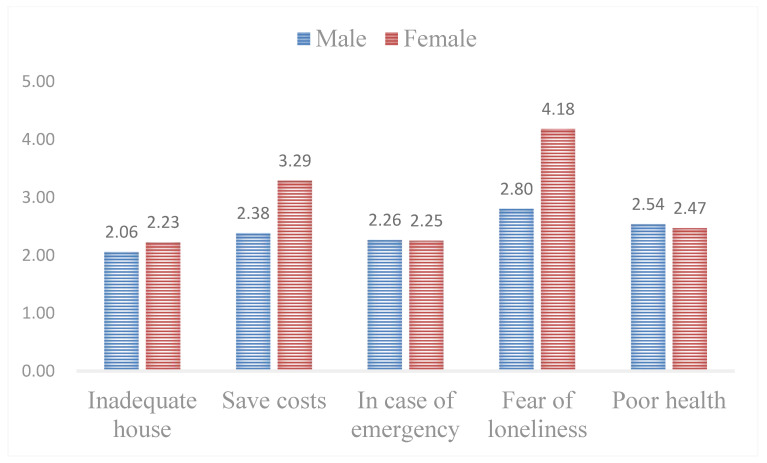
Intergenerational factors in different genders.

Gender roles in the nucleus of the family institution indicate the distribution of tasks [57], thus gender differences in intergenerational factors influencing household cohabitation should not be ignored. By calculating the average of the factor scores, significant gender differences were seen in intergenerational factors (Figure 4).

Inadequate housing and poor health are common considerations in intergenerational household cohabitation. Yet women are more concerned with saving costs and fear of loneliness. Meanwhile, “in case of emergency” matters to everybody involved in intergenerational household cohabitation, not just for oneself, but for the whole family (Figure 4). According to factor classification in Table 3, financial support and daily care support are common considerations in intergenerational household cohabitation, and women are more concerned about emotional support.

(3) Variation in groups of all genders and ages

**Figure 5:**
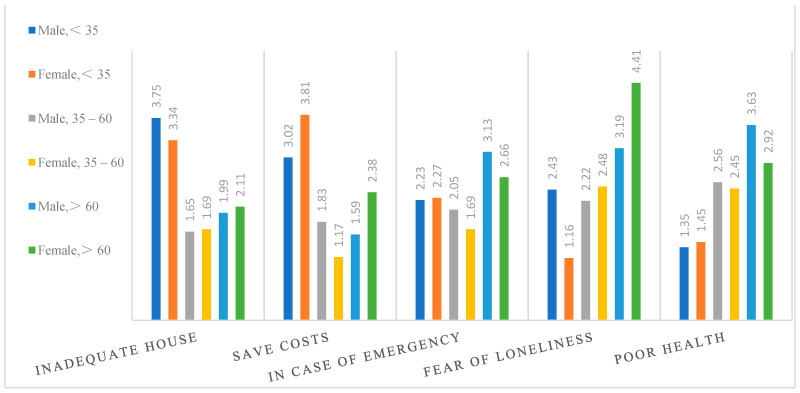
Intergenerational factors in groups of all genders and ages.

Via calculating the average of the factor scores, variation and differences in intergenerational factors in groups of all genders and ages is shown in Figure 5.

Inadequate housing has the greatest effect in individuals under 35, affecting men more than women, and its effect decreases with age. Saving costs is also an important factor in intergenerational household cohabitation for individuals under 35, and its effect has also been seen in a group of women over 60 years. “In case of emergency” is significant for all genders and ages, especially for individuals aged over 60 years, who, when they are ill or suffering from an emergency, need help from adult children. Fear of loneliness counts more for individuals aged over 60 years; affected by traditional Chinese culture of “family fun between the older and younger generations who are related by blood”, elders prefer intergenerational household cohabitation. Interestingly, fear of loneliness only has a very weak effect on women under 35, thus leading to a stark contrast between women under 35 and over 60. These two groups constitute the most irreconcilable contradiction (mother-in-law and daughter-in-law contradiction) in China. We found in the interviews that women under 35 pay little attention to fear of loneliness, due to worrying about conflicts between mother-in-law and daughter-in-law and preferring a relatively undisturbed life. Poor health is important for individuals aged over 60 years, and the effect on men over 60 is even more pronounced. One possible explanation given in the study is that men are more independent and less willing to live in intergenerational household cohabitation, but if they are in poor health they will choose intergenerational household cohabitation, so that they can obtain care from their children.

We come to the conclusion that daily care support is a common factor in intergenerational household cohabitation, financial support is comparatively important for individuals under 35, and females attach more importance to emotional support.

## 5. Discussion

Throughout Chinese history, intergenerational support has evolved from moral values and beliefs. Respecting and taking care of elders is ingrained in traditional Chinese families. Commonly, sons and daughters-in-law are supposed to provide material support to their elderly parents, since their daughters will traditionally switch to their husbands’ family. Older sons are most likely to reside with their parents; however, parents also frequently live with younger sons. Parents with no sons have no alternative but to live with their married daughters [58].

During the Chinese transition period, more than 30 years of ultra-high-speed economic development and the reform of the housing supply system led to an explicit and significant differentiation of intergenerational household cohabitation structures. Based on the real-life situation of urban families, and some special conditions, such as the one-child policy the one-child family is still the norm in Chinese cities), intergenerational relationships within urban families in Chengdu have changed.

Inadequate housing is the main factor in intergenerational household cohabitation. Housing property rights are what people have been struggling for in urban China and are still deeply rooted in traditional Chinese economy and culture. In urban China, the house is not only the place where one lives, but is also a symbol of status. Where there is no retirement home in the local area, intergenerational household cohabitation represents an alternative. Simultaneously, intergenerational household cohabitation can effectively reduce family spending; indeed, grandparents would often rather give their grandchildren pocket money than pay for a nursing home. One respondent to our survey stated, “I am old. Why do I want to live in an ice-cold nursing home? I just live with my children and grandson.” In terms of having access to care in case of an emergency, intergenerational household cohabitation is also an optimal choice, which is again influenced by the values of traditional Chinese culture. Parents are expected to take care of their children when parents are healthy, so that they can be supported in turn by their children as they get older. This investigation proves that there is a rising tendency towards household cohabitation with increasing age. On the other hand, with increasing age, elders worry about being forgotten by their children. Physical conditions are also important with regard to intergenerational household cohabitation. Moreover, previous studies have manifested that male elders prefer intergenerational cohabitation more than elder women. Moreover, widows are more willing than widowers to choose intergenerational cohabitation.

This study is subject to several limitations. First, the social and economic effect of household cohabitation has not been analyzed. Second, the impact of household cohabitation on urban spatial restructuring has not been explored. However, despite the deficiencies noted above, this paper makes a significant contribution to the field. Intergenerational support for household cohabitation in urban China can be summed up systematically. However, the intriguing results here may inspire more projects about families living with elders. There is no one-size-fits-all pattern, but intergenerational factors should be taken into account, as this will improve quality of life for the family.

## 6. Conclusions

This preliminary research effort is one of the first empirical studies on intergenerational support and household cohabitation in urban families in China. Subsequent studies should focus on how intergenerational support is affected by family composition. Although this paper is subject to some limitations (as outlined above), it provides a basis for future studies, and supports existing studies regarding intergenerational support for household cohabitation in urban China.

## Figures and Tables

**Table 1:** Sample distribution.

Age	Elder Live with Married Children	Ratio (%)	Elder Live with Unmarried Children	Ratio (%)	Elder Live with Grandchildren	Ratio (%)
<25	25	1.01	92	3.73	1	0.04
25–34	354	14.36	192	7.79	6	0.24
35–44	303	12.29	55	2.23	91	3.69
45–54	271	10.99	42	1.70	84	3.41
55–64	183	7.42	37	1.50	108	4.38
65–74	187	7.59	31	1.26	181	7.34
75–84	95	3.85	24	0.97	85	3.45
85<	6	0.24	3	0.12	9	0.37

**Table 2:** Changes in household cohabitation from 2000 to 2010 (Ratio, %).

District	One Elder	Two Elders	More Than Two Elders
Jinjiang	−10.94	−6.97	−0.17
Qingyan	−11.24	−9.04	−0.10
Jinniu	−7.56	−5.37	−0.07
Wuhou	−12.52	−9.37	−0.11
Chenghua	−9.40	−7.19	−0.08

**Table 3:** Intergenerational factors in household cohabitation.

Financial Support	Daily Care Support	Emotional Support
Inadequate house	In case of emergency	Fear of loneliness
Save costs	Doing the housework	Enjoyment of living with a big family
	Poor health	Care of grandchildren

**Table 4:** Characteristics of sample (N = 2465).

Factors	Mean	Standard Deviation	Range
Inadequate house	2.136	1.343	1–5
Save costs	2.796	1.442	1–5
In case of emergency	2.259	1.486	1–5
Doing the housework	1.845	1.367	1–5
Poor health	2.475	1.062	1–5
Fear of loneliness	2.562	1.604	1–5
Enjoyment of living with a big family	2.303	1.436	1–5
Care of grandchildren	2.505	1.463	1–5

**Table 5:** Multiple logistic regression of intergenerational support in household cohabitation (N = 2465).

Variables	Elderly with Grandchildren				Elderly with Unmarried Children			
	B	Sig.	S.E.	Exp (B)	B	Sig.	S.E.	Exp (B)
Inadequate house	−0.861	***	0.159	0.423	0.612	***	0.148	0.542
Save costs	0.346	*	0.177	0.708	0.717	***	0.160	2.049
In case of emergency	−0.518	***	0.132	0.595	0.221	*	0.121	0.802
Enjoyment of living with a big family					−0.376	**	0.155	0.686
Doing the housework					1.087	***	0.193	2.966
Fear of loneliness	0.600	***	0.141	0.549	−0.300	**	0.128	0.740
Care of grandchildren	0.471	***	0.156	0.624				
Poor health	0.418	***	0.134	0.658	0.239	*	0.127	0.787
Constant	7.66	***	1.119		1.593	***	1.001	
−2 log likelihood					530.137			
Chi-square					407.54			
Df					16			
Significance					0.000			
Nagelkerke					0.655			
McFadden					0.435			

Notes: Blank cells indicate that the result was not significant. * = *p* < 0.10, ** = *p* < 0.05, *** = *p* < 0.01.

## Data Availability

Not applicable.
